# Study of the Etiology of Acute Respiratory Infections in Children Under 5 Years at the Dr. Agostinho Neto Hospital, Praia, Santiago Island, Cabo Verde

**DOI:** 10.3389/fped.2021.716351

**Published:** 2021-09-28

**Authors:** Wilson Correia, Roberto Dorta-Guerra, Mitza Sanches, Carmen de Jesús Borges Almeida Semedo, Basilio Valladares, Isabel Inês M. de Pina-Araújo, Emma Carmelo

**Affiliations:** ^1^Instituto Universitario de Enfermedades Tropicales y Salud Pública de Canarias, Universidad de La Laguna, La Laguna, Spain; ^2^Departamento de Matemáticas, Estadística e Investigación Operativa, Facultad de Ciencias, Universidad de La Laguna, La Laguna, Spain; ^3^Hospital Dr. Agostinho Neto, Ministry of Health and Social Security of Cabo Verde, Praia, Cabo Verde; ^4^Departamento de Obstetricia y Ginecología, Pediatría, Medicina Preventiva y Salud Pública, Toxicología, Medicina Legal y Forense y Parasitología, Universidad de La Laguna, La Laguna, Spain; ^5^Faculty of Sciences and Technology, University of Cabo Verde, Praia, Cabo Verde; ^6^Red de Investigación Colaborativa en Enfermedades Tropicales (RICET), Madrid, Spain

**Keywords:** children, acute respiratory infections, ARI, molecular diagnostics, FilmArray, Cabo Verde, pediatric infections

## Abstract

**Background:** Acute respiratory infections are one of the major causes of morbidity and mortality in children under 5 years in developing countries and are a challenge for the health system of these countries. In Cabo Verde, despite the lack of recent studies, data indicate that it affects thousands of children, being the fourth leading cause of infant mortality in 2013. The aim of this study was to identify and describe the etiological agents associated with acute respiratory tract infections in children under 5 years old, and their associated risk factors, such as clinical symptoms or socio-demographic characteristics.

**Methods:** Naso-pharyngeal samples were collected from children under 5 years attending at Dr. Agostinho Neto Hospital (Praia, Santiago Island, Cabo Verde) with suspected ARI at different time-points during 2019. Samples were analyzed using *FilmArray*® *Respiratory Panel v. 2.0 Plus* to identify etiological agents of ARI. A questionnaire with socio-demographic information was also collected for each participant. Data analyses were carried out using the IBM SPSS version 25 (IBM Corporation, Armonk, NY) and R 3.5.1 statistical software.

**Results:** A total of 129 naso-pharyngeal samples were included in the study. Seventeen different etiologic agents of respiratory infections were identified. HRV/EV was the most frequent agent detected, followed by FluA H3 and RSV. Coinfection with two or more pathogens was detected in up to 20% of positive samples. The results were analyzed in terms of age-group, sex, period of the year and other social and demographic factors.

**Conclusion:** Viruses are the main causative agents of ARI in children <5 years attending at the pediatrics service at the Dr. Agostinho Neto Hospital in Praia city, Santiago Island, Cabo Verde. Some factors are described in this study as statistically associated with the presence of an infectious agent, such as having one or more children sharing the bedroom with an adult and the presence of some clinical symptoms. The data addresses the need for studies on respiratory tract infections in Cabo Verde.

## Introduction

Acute respiratory infections (ARI) are defined as infections in the respiratory tract (lower and/or upper), resulting in obstruction of the air passage at the nasal and/or bronchial system, causing a spectrum of manifestations, from acute symptoms, like common colds, to more serious conditions such as pneumonia or lung collapse (1). ARI often constitutes a medical emergency, because it directly affects tissue oxygenation, driving to complications in children, with poor outcomes including increased morbidity and mortality. It is not uncommon that ARI requires intensive care, permanent evaluation, as well as quick and resolutive interventions (2, 3).

Despite the usually benign nature of the infection, ARI is an enormous economic burden on society in terms of visits to doctors and other health-care providers, treatments, and absences from work, school and/or day care (4).

Among ARI, infections of the upper respiratory tract are the most frequent, but the majority of respiratory deaths are attributed to acute lower respiratory infections (5, 6). Both bacteria and viruses have been identified as the agents of ARI, however, it is known that 90% of these infections have viral origin (2, 7). In children with ARI, respiratory syncytial virus (RSV), influenza virus types A and B (Flu A and Flu B), adenovirus (ADV), parainfluenza virus (PIV), human metapneumovirus (hMPV) and human rhinovirus/enterovirus (HRV/EV) are the most frequently detected viruses (7, 8). Other viruses such as human bocavirus (hBoVs), and mainly severe acute respiratory syndrome coronavirus (SARS-CoV), middle east respiratory syndrome coronavirus (MERS-CoV), and more recently a new type of coronavirus (SARS-CoV-2), the causative agent of COVID-19 respiratory infection worldwide, have been described as responsible for more severe symptoms like respiratory distress syndrome (9, 10). Bacteria such as *Streptococcus pneumoniae, Staphylococcus aureus* and *Klebsiella pneumoniae* are less frequently reported (6, 7, 11).

The clinical manifestations of respiratory infections caused by these agents are similar in most cases, including cough, fever ≥ 38°C, headache and/or difficult breathing, among others. On the other hand, the clinical spectrum is variable, ranging from mild infections, which can be treated on an outpatient basis, to more serious forms that require hospitalization, particularly in patients with cardiopathy or metabolic diseases (6, 12–15). The early detection of the potential causative agents of ARI is essential for appropriate treatment, helping to reduce the overuse of antibiotics therapy, and prevent outbreaks or reinfection. In this context, polymerase chain reaction (PCR) assays have shown to be a sensitive and specific tool for detection of these agents, particularly the multiplex-PCR assays, enabling the detection of multiple targets in a single clinical specimen (9).

ARI are the main cause of morbidity and mortality worldwide, especially in children under 5 years (7). It is responsible for more than 12 million hospital admissions and 1.9 to 2.2 million deaths in children each year, 70% occurring in Africa and Southeast Asia (6, 16, 17). Studies show that the etiological agents of ARIs are geographically diverse and are associated with the epidemic status of each ARIs and climate conditions (18). Therefore, data on the epidemiology of ARI cases are very important to develop control and prevention strategies (11).

Archipelago of Cabo Verde is located off the West African coast, 500 km away from Senegal. With a total of 4,033 km^2^ and 544,081 residents, Cabo Verde has a young population, with 47% of people under 24 years-old and only 6% of over 65 years old (19, 20). Cabo Verde is facing demographic and epidemiological changes, as well as an increasingly demanding population. The country needs to address “old” challenges, such as controlling transmissible diseases, and at the same time tackle emerging needs such as responding to the increasing prevalence of non-transmissible diseases. To keep control of this challenges, the country has provided a new national health plan, the National Health Development Plan 2017–2021, including 8 strategies of development and operational initiatives to improve the health of the population of Cabo Verde (20).

According to WHO, in 2013 ARI was considered the fourth cause of mortality in children under 5 years in Cabo Verde (21). Between 2014 and 2018, ARI was the condition showing the highest notification in children under 5 years with an average of 31,238 cases per year (22). Despite the high notification rate, there is little scientific literature describing the etiological agents of ARI and the associated risk factors in Cabo Verde. Only one research project was conducted between 2009 and 2010 in Senegal, Cabo Verde, Mauritania and Guinea, revealing the involvement of the influenza virus A and B in complications of the respiratory tract in samples collected from patients seeking health care in those countries (23). Of 3,155 samples tested, 911 (28.9%) were positive for influenza virus, with influenza A being the most prevalent virus detected, and patients aged between 5 and 14 years and <5 years were the most affected (72/422, 17.1%; 262/1642; 16.2%, respectively) (23).

Determining the etiological agents of ARI in Cabo Verde is fundamental not only for surveillance, for diagnostic and therapeutic purposes, but also to illustrate the relationships between ARI in children and local demographic, socioeconomic and environmental factors, in order to contribute to defining public health policies regarding ARI. The aim of this study was to identify the etiological agents associated with acute respiratory tract infections in children under 5 years at Dr. Agostinho Neto Hospital (Praia, Santiago Island, Cabo Verde) and the clinical symptoms associated with this infection. In parallel, the distribution of ARI by age, sex, season, geographic location and their associated risk factors was described.

## Materials and Methods

### Study Design and Data Collection

**Figure 1:**
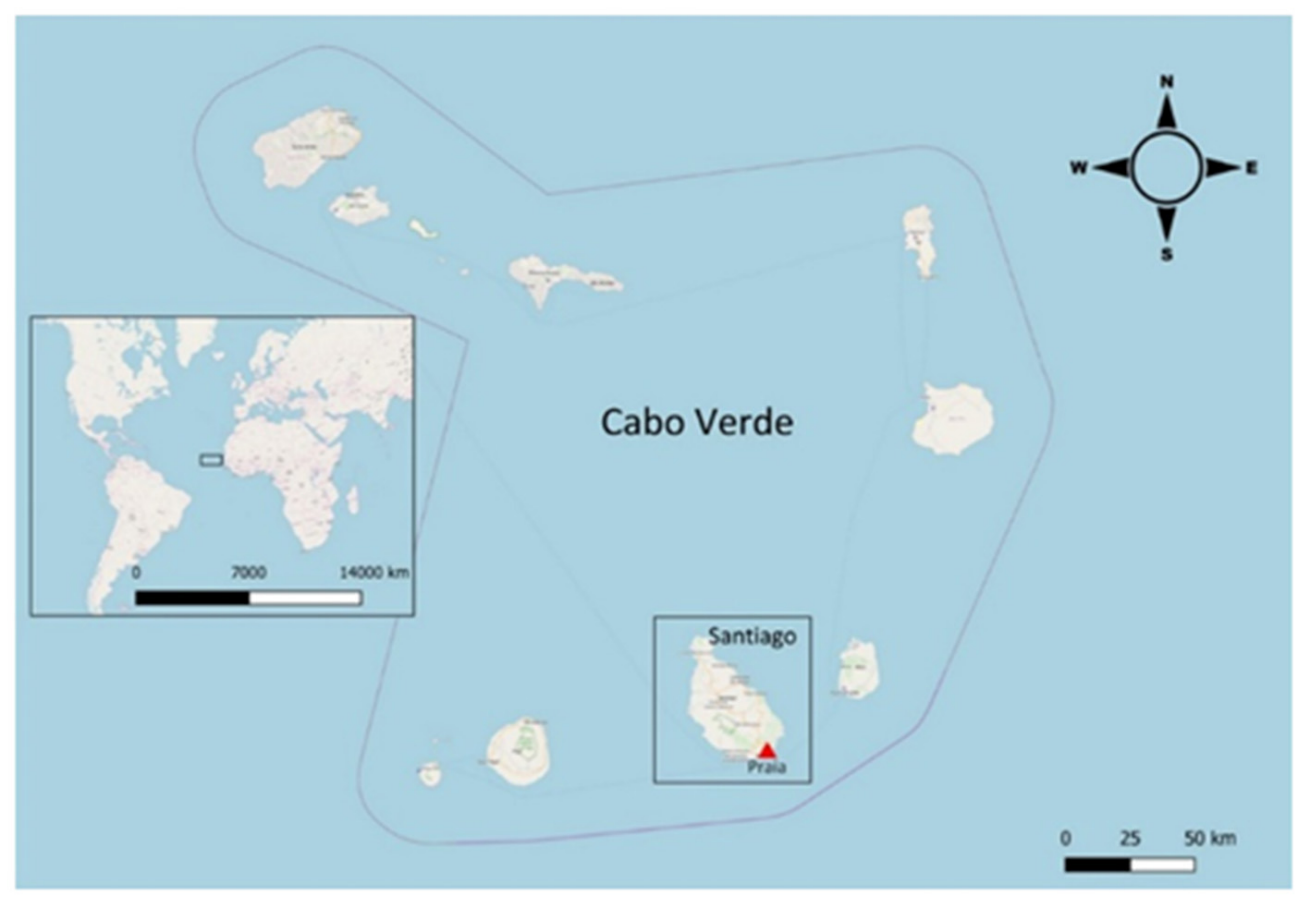
Location of Praia in Santiago Island (Cabo Verde). Map modified with QGIS 3.8.0-Zanzibar (www.qgis.org) from OpenStreetMap.org. OpenStreetMap® is open data, licensed under the Open Data Commons Open Database License (ODbL) by the OpenStreetMap Foundation (OSMF).

We collected cross-sectional data in January, May and November 2019 of children under 5 years of age attended at the Pediatrics' emergency and ambulatory service at Hospital Dr. Agostinho Neto (HAN) in Praia city, Santiago Island, Archipelago of Cabo Verde with suspected acute respiratory infection (ARI) (Figure 1).

To be included in the study, children were identified by clinicians and must have had three or more typical symptoms of ARI (cough, nasal obstruction, chest pain, headache, difficult breathing, conjunctivitis and/or fever ≥38°C), starting at least 3–7 days before, and not under any treatment.

These inclusion criteria were defined by Fitzner et al. (15). In some cases, according to medical recommendations, children with less than three typical symptoms of ARI were included in the study.

All samples were accompanied by a questionnaire, containing child socio-demographic and clinical data provided by the assisting medical professionals as well as parents and/or guardian. Specimens were naso-pharyngeal swabs (NPS) collected in 3 ml of Viral Transport Medium (VTM) (Delta lab, Barcelona, Spain), according to Baden et al. (24). Samples were stored at −80°C until transport to the laboratory of the Instituto Universitario de Enfermedades Tropicales y Salud Pública de Canarias, Universidad de La Laguna (IUETSPC/ULL) in Tenerife, Canary Islands.

### FilmArray Testing

The detection of the virus and bacteria considered etiological agents of ARI was performed using *FilmArray*® *Respiratory Panel v.2.0 Plus* (FARP–FilmArray® Respiratory Panel BioFire Diagnostics LLC 390; Wakara Way Salt Lake City, UT, USA). *FilmArray RP2plus* is a test based on multiplex PCR (25) designed for the simultaneous detection and identification of nucleic acids of 18 respiratory viruses and 4 bacteria (26). The viruses are Adenovirus (ADV), Coronavirus 229E (CoV 229E), Coronavirus HKU1 (CoV HKU1), Coronavirus OC43 (CoV OC43), Coronavirus NL63 (CoV NL63), Human Rhinovirus/Enterovirus (HRV/EV), Human Metapneumovirus (hMPV), Influenza A (FluA), Influenza A H1 2009 (Flu A H1 2009), Influenza A H3 (Flu A H3), Influenza A H1 (Flu A H1), Influenza B (Flu B), Parainfluenza Virus 1 (PIV 1), Parainfluenza Virus 2 (PIV 2), Parainfluenza Virus 3 (PIV 3), Parainfluenza Virus 4 (PIV 4), Respiratory Syncytial Virus (RSV), and Middle East Respiratory Syndrome (MERS-CoV). The bacteria are *Chlamydophila pneumoniae, Mycoplasma pneumoniae, Bordetella parapertussis* and *Bordetella pertussis* (26, 27). A fraction of 300 μl of each sample was subjected to *FilmArray RP2plus* testing according to the manufacturer's instructions (available in: www.biofiredx.com).

### Statistical Analysis

Data analyses were carried out using the IBM SPSS version 25 (IBM Corporation, Armonk, NY) and R 3.5.1 statistical software. Results are presented as means ± standard deviations (SD) for continuous data and proportions (prevalence) for categorical data. Ninety-five percent confidence intervals for prevalence using the approximate or exact method, as appropriate, were included.

Chi-square test or Fisher's exact test, as appropriate, were assessed to study the associations between the presence of pathogens and some sociodemographic variables. Results with *p* < 0.05 were considered statistically significant. To determine predictor variables for ARI, a binary logistic regression model was fitted and variables at a *p* < 0.2 during the bi-variate analysis were included in the multivariable analysis. All assumptions for binary logistic regression were checked. Finally, variables found to be significant at a *p* < 0.05 in the final model were declared as predictors. Crude odds ratios (COR) and adjusted odds ratios (AOR) with 95% confidence interval were reported. Hosmer and Lemeshow goodness-of-fit test (*p* > 0.05) was used to check model fitness.

## Results

### Demographic Characteristics

A total of 129 naso-pharyngeal *swabs* from children <5 years old with suspected ARI were collected and analyzed during the study period. In detail, 60 (46.5%) of them belong to children <1 years of age and 69 (53.5%) between 1 and 4 years old. The sex distribution was 62 (48.1%) female and 67 (51.9%) male samples. Sampling was performed in three different periods in 2019, 35 (27.1%) in January, 46 (35.7%) in May and 48 (37.2%) in November. Most of the patients (88.4%, 114/129) came from Praia city, and in two patients this information is lacking (Table 1). According to the clinical signs and symptoms, most patients enrolled in this study presented with nasal obstruction (119, 92.2%), cough (112, 86.9%) and fever ≥38°C (84, 65.1%). Difficult breathing (28, 21.7%), conjunctivitis (6, 4.7%), chest pain (4, 3.1%), and headache (3, 2.3%) were also reported. A total of 63 (48.8%) patients enrolled in this study presented the first symptoms between 6 and 7 days before attending HAN, 40 (31.0%) at 3–5 days and 26 (20.2%) presented first symptoms 1–2 days before seeking medical attention. Most (88.4%, 114/129) of the children lived in Praia, the capital city of Santiago Island that can be divided in 4 geographic areas: North of Praia, West of Praia, South of Praia, and East of Praia. Positive samples were distributed among all areas, showing no clear geographical pattern (Table 1).

**Table 1:** Frequency (%) of respiratory pathogens by age, sex, study period and geographic areas in Praia, during 2019.

	**Prevalences**		**Age**, **+/n (%)**			**Sex**, **+/n (%)**			**Study Period**, **+/n (%)**				**Geographic areas in Praia**, **+/n (%)**					
**Pathogen**	**+/n (%)**	**(95% CI)**	** <1 year**	**1–4 years**	***Sig*.**	**Female**	**Male**	***Sig*.**	**January**	**May**	**November**	***Sig*.**	**North**	**West**	**South**	**East**	**Other**	***Sig*.**
ADV	12/129 (9.3)	(4.9–15.7)	6/60 (10.0)	6/69 (8.7)	n.s.	9/62 (14.5)	3/67 (4.5)	n.s.	2/35 (5.7)	6/46 (13.0)	4/48 (8.3)	n.s.	4/31 (12.9)	3/22 (13.6)	2/44 (4.5)	2/17 (11.8)	0/13 (0.0)	n.s.
CoV 229E	1/129 (0.8)	(0.0–4.2)	1/60 (1.7)	0/69 (0.0)	n.s.	0/62 (0.0)	1/67 (1.5)	n.s.	1/35 (2.9)	0/46 (0.0)	0/48 (0.0)	n.s.	0/31 (0.0)	0/22 (0.0)	0/44 (0.0)	0/17 (0.0)	0/13 (0.0)	n.s.
CoV HKU1	1/129 (0.8)	(0.0–4.2)	1/60 (1.7)	0/69 (0.0)	n.s.	1/62 (1.6)	0/67 (0.0)	n.s.	0/35 (0.0)	1/46 (2.2)	0/48 (0.0)	n.s.	0/31 (0.0)	1/22 (4.5)	0/44 (0.0)	0/17 (0.0)	0/13 (0.0)	n.s.
CoV OC43	4/129 (3.1)	(0.9–7.7)	2/60 (3.3)	2/69 (2.9)	n.s.	1/62 (1.6)	3/67 (4.5)	n.s.	1/35 (2.9)	2/46 (4.3)	1/48 (2.1)	n.s.	2/31 (6.5)	0/22 (0.0)	1/44 (2.3)	0/17 (0.0)	1/13 (7.7)	n.s.
CoV NL63	2/129 (1.6)	(0.2–5.5)	1/60 (1.7)	1/69 (1.4)	n.s.	0/62 (0.0)	2/67 (3.0)	n.s.	0/35 (0.0)	0/46 (0.0)	2/48 (4.2)	n.s.	1/31 (3.2)	0/22 (0.0)	0/44 (0.0)	0/17 (0.0)	1/13 (7.7)	n.s.
HRV/EV	51/129 (39.5)	(31.0–48.5)	28/60 (46.7)	23/69 (33.3)	n.s.	25/62 (40.3)	26/67 (38.8)	n.s.	9/35 (25.7)	24/46 (52.2)	18/48 (37.5)	n.s.	11/31 (35.5)	10/22 (45.5)	19/44 (43.2)	6/17 (35.3)	4/13 (30.8)	n.s.
hMPV	9/129 (7.0)	(3.2–12.8)	6/60 (10.0)	3/69 (4.3)	n.s.	4/62 (6.5)	5/67 (7.5)	n.s.	1/35 (2.9)	8/46 (17.4)	0/48 (0.0)	0.001	3/31 (9.7)	1/22 (4.5)	2/44 (4.5)	1/17 (5.9)	2/13 (15.4)	n.s.
RSV	13/129 (10.1)	(5.5–16.6)	6/60 (10.0)	7/69 (10.1)	n.s.	7/62 (11.3)	6/67 (9.0)	n.s.	0/35 (0.0)	1/46 (2.2)	12/48 (25.0)	<0.001	4/31 (12.9)	2/22 (9.1)	3/44 (6.8)	2/17 (11.8)	1/13 (7.7)	n.s.
FluA H1-2009	1/129 (0.8)	(0.0–4.2)	1/60 (1.7)	0/69 (0.0)	n.s.	1/62 (1.6)	0/67 (0.0)	n.s.	1/35 (2.9)	0/46 (0.0)	0/48 (0.0)	n.s.	0/31 (0.0)	1/22 (4.5)	0/44 (0.0)	0/17 (0.0)	0/13 (0.0)	n.s.
FluA H3	18/129 (14.0)	(8.5–21.2)	4/60 (6.7)	14/69 (20.3)	0.049	8/62 (12.9)	10/67 (14.9)	n.s.	6/35 (17.1)	0/46 (0.0)	12/48 (25.0)	<0.001	6/31 (19.4)	1/22 (4.5)	8/44 (18.2)	2/17 (11.8)	1/13 (7.7)	n.s.
FluB	4/129 (3.1)	(0.9–7.7)	1/60 (1.7)	3/69 (4.3)	n.s.	1/62 (1.6)	3/67 (4.5)	n.s.	0/35 (0.0)	0/46 (0.0)	4/48 (8.3)	0.037	1/31 (3.2)	0/22 (0.0)	3/44 (6.8)	0/17 (0.0)	0/13 (0.0)	n.s.
PIV 1	1/129 (0.8)	(0.0–4.2)	0/60 (0.0)	1/69 (1.4)	n.s.	1/62 (1.6)	0/67 (0.0)	n.s.	1/35 (2.9)	0/46 (0.0)	0/48 (0.0)	n.s.	0/31 (0.0)	0/22 (0.0)	0/44 (0.0)	1/17 (5.9)	0/13 (0.0)	n.s.
PIV 2	1/129 (0.8)	(0.0–4.2)	1/60 (1.7)	0/69 (0.0)	n.s.	0/62 (0.0)	1/67 (1.5)	n.s.	1/35 (2.9)	0/46 (0.0)	0/48 (0.0)	n.s.	0/31 (0.0)	0/22 (0.0)	1/44 (2.3)	0/17 (0.0)	0/13 (0.0)	n.s.
PIV 3	3/129 (2.3)	(0.5–6.6)	3/60 (5.0)	0/69 (0.0)	n.s.	2/62 (3.2)	1/67 (1.5)	n.s.	2/35 (5.7)	1/46 (2.2)	0/48 (0.0)	n.s.	1/31 (3.2)	1/22 (4.5)	1/44 (2.3)	0/17 (0.0)	0/13 (0.0)	n.s.
PIV 4	1/129 (0.8)	(0.0–4.2)	0/60 (0.0)	1/69 (1.4)	n.s.	1/62 (1.6)	0/67 (0.0)	n.s.	0/35 (0.0)	0/46 (0.0)	1/48 (2.1)	n.s.	0/31 (0.0)	0/22 (0.0)	0/44 (0.0)	0/17 (0.0)	1/13 (7.7)	n.s.
*C. pneumoniae*	3/129 (2.3)	(0.5–6.6)	1/60 (1.7)	2/69 (2.9)	n.s.	0/62 (0.0)	3/67 (4.5)	n.s.	0/35 (0.0)	2/46 (4.3)	1/48 (2.1)	n.s.	1/31 (3.2)	1/22 (4.5)	1/44 (2.3)	0/17 (0.0)	0/13 (0.0)	n.s.
*M. pneumoniae*	1/129 (0.8)	(0.0–4.2)	0/60 (0.0)	1/69 (1.4)	n.s.	0/62 (0.0)	1/67 (1.5)	n.s.	0/35 (0.0)	1/46 (2.2)	0/48 (0.0)	n.s.	1/31 (3.2)	0/22 (0.0)	0/44 (0.0)	0/17 (0.0)	0/13 (0.0)	n.s.
≥1 pathogen	102/129 (79.1)	(71.0–85.7)	51/60 (85.0)	51/69 (73.9)	n.s.	49/62 (79.0)	53/67 (79.1)	n.s.	23/35 (65.7)	36/46 (78.3)	43/48 (89.6)	0.030	27/31 (87.1)	16/22 (72.7)	36/44 (81.8)	13/17 (76.5)	8/13 (61.5)	n.s.

*ADV, Adenovirus; CoV 229E, Coronavirus 229E; CoV HKU1, Coronavirus HKU1; CoV OC43, Coronavirus OC43; CoV NL63, Coronavirus NL63; HRV/EV, Human Rhinovirus/Enterovirus; hMPV, Human Metapneumovirus; RSV, Respiratory Syncytial Virus; FluA H1 2009, Influenza A H1 2009; FluA H3, Influenza A H3; FluB, Influenza B; PIV 1, Parainfluenza virus 1; PIV 2, Parainfluenza Virus 2; PIV 3, Parainflueza Virus3; PIV 4, Parainfluenza Virus 4; C. pneumoniae, Chamydophila pneumoniae; M. pneumoniae, Mycoplasma pneumoniae*.

### Respiratory Pathogens Causing ARI in Children Under 5 Years Old in Cabo Verde

**Figure 2:**
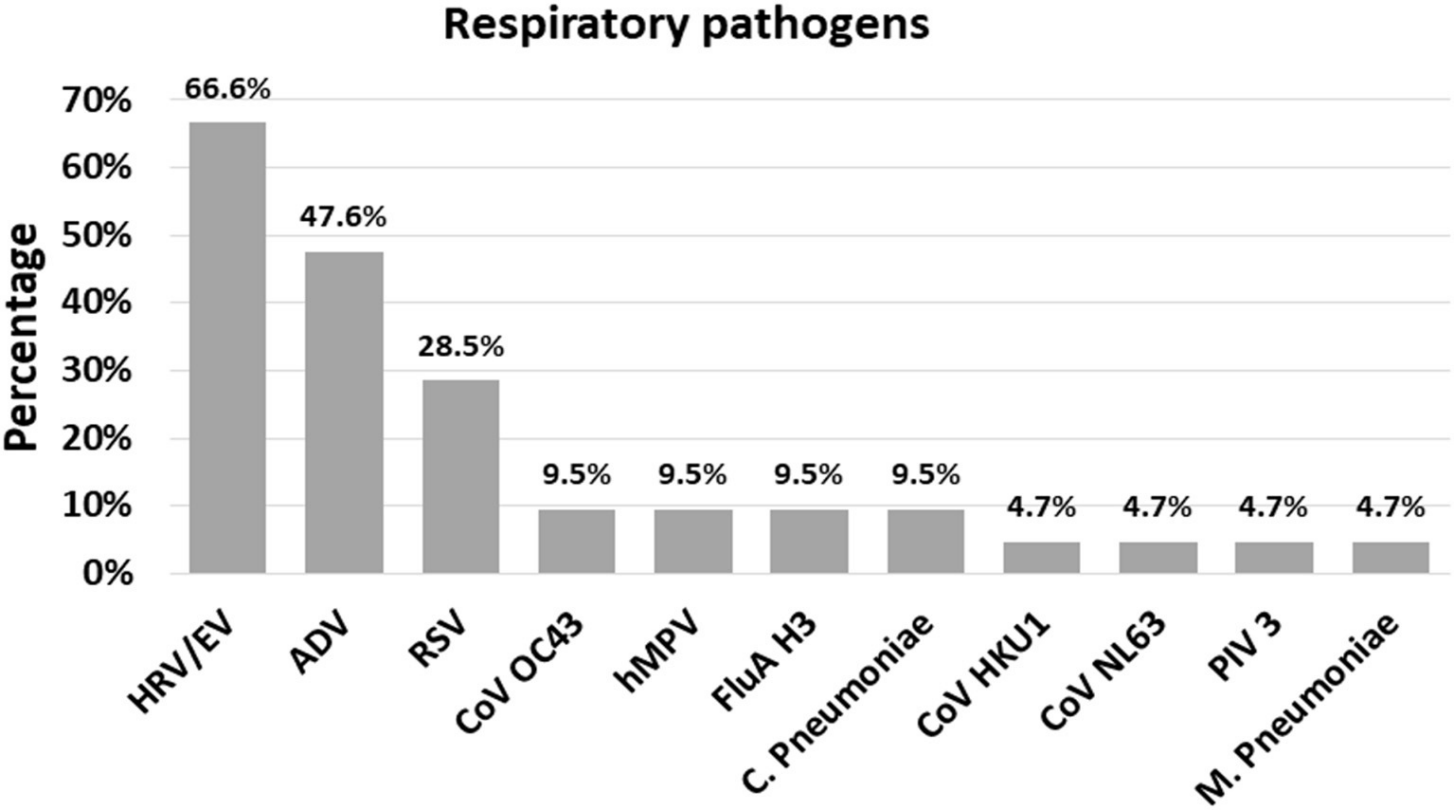
The frequency (%) of each respiratory pathogen in the context of a co-infection. ADV, Adenovirus; CoV HKU1, Coronavirus HKU1; CoV OC43, Coronavirus OC43; CoV NL63, Coronavirus NL63; HRV/EV, Human Rhinovirus/Enterovirus; hMPV, Human Metapneumovirus; RSV, Respiratory Syncytial Virus; FluA H3, Influenza A H3; PIV 3, Parainflueza Virus 3; C. Pneumoniae, *Chamydophila pneumoniae*; M. Pneumoniae, *Mycoplasma pneumoniae*.

Among the 129 naso-pharyngeal samples tested, 102 (79.1%) were positive for one or more of the pathogens detected in the *Panel*. HRV/EV was the most frequent respiratory pathogen (39.5%), followed by FluA H3 (14.0%) and RSV (10.1%; Table 1). The detection rates for all other pathogens tested were below 10% (Table 1). A single respiratory pathogen was detected in 81 (62.8%) positive samples, and multiple pathogens were detected in 21 of them (20.6%; Table 2), generally combinations of HRV/EV (14/21, 66.6%), ADV (10/21, 47.6%), or RSV (6/21, 28.5%) with a different infectious agent (Figure 2, Table 2).

**Table 2:** Reported cases of respiratory pathogens co-infection in the study population.

**Presentation**		**+/n (%)**
Single pathogens infection		81/102 (79.4)
Co-infection		21 /102 (20.6)
2 pathogens	ADV+HRV/EV	6/21 (28.5)
	HRV/EV+RSV	4/21 (19.0)
	ADV+CoV HKU1	1/21 (4.7)
	ADV+hMPV	1/21 (4.7)
	ADV+RSV	1/21 (4.7)
	CoV OC43+HRV/EV	1/21 (4.7)
	CoV OC43+CoV NL63	1/21 (4.7)
	FluA H3+ *C. pneumoniae*	1/21 (4.7)
	HRV/EV+FluA H3	1/21 (4.7)
	HRV/EV+hMPV	1/21 (4.7)
	HRV/EV+PIV 3	1/21 (4.7)
	*C. pneumoniae+M. pneumoniae*	1/21 (4.7)
3 pathogens	ADV+HRV/EV+RVS	1/21 (4.7)

*ADV, Adenovirus; CoV HKU1, Coronavirus HKU1; CoV OC43, Coronavirus OC43; CoV NL63, Coronavirus NL63; HRV/EV, Human Rhinovirus/Enterovirus; hMPV, Human Metapneumovirus; RSV, Respiratory Syncytial Virus; FluA H3, Influenza A H3; PIV 3, Parainfluenza Virus 3; C. pneumoniae, Chamydophila pneumoniae; M. pneumoniae, Mycoplasma pneumoniae*.

The combination of three respiratory pathogens was found in a single sample, representing 4.8% of co-infections. The most frequent finding in terms of co-infection was ADV + HRV/EV viruses, with six cases (28.5%), followed by HRV/EV + RSV (19.0%; Table 2).

Age clustering revealed a balanced positivity rate, with 85.0% positives in the <1 year old age group and 73.9% in the 1–4 years old (Table 1). Besides, most pathogens were detected at similar rates in both age groups. In contrast, FluA H 3 was most prevalent in the 1–4 year old group (*p* = 0.049, Table 1), suggesting an influence of age for this infection. Regarding sex, no statistically significant differences were observed between boys' and girls' infection rates (Table 1). It is interesting the higher frequency of ADV infections in the female group (14.5%), although no statistically significant differences were observed (Table 1).

Sampling was performed in three different periods along 2019 (January, May, and November) in order to identify possible seasonal distribution of this collection of pathogens. The highest sample positive rate was detected in November (89.6%), followed by May (78.3%) and January (65.7%; Table 1). Out of the 17 pathogens detected, four showed statistically significant differences among the three sampling periods, being FluA H3, FluB and RSV most frequent in November and hMPV in May (Table 1).

Only two bacterial pathogens were found in our study: *C. pneumoniae* was detected in May and November (4.3%, 2/46; 2.1%, 1/48, respectively), while *M. pneumoniae* only in May (2.2%, 1/46), all of them in low frequency.

The association between clinical presentation and ARI was also evaluated (Table 3). The bivariable analysis revealed that the days from the symptom onset, the presence of three or more typical symptoms of ARI, and particularly the presence of nasal obstruction, chest pain, conjunctivitis, or cough, were variables with a *p* < 0.2, and therefore were included in the multivariable logistic regression model. The forward stepwise binary logistic regression model accounted for 14.5% (Nagelkerke *R*^2^ = 0.145) of the variance in prevalence. The presence of nasal obstruction and chest pain were significantly associated with ARI among children <5 years in the final model. The adjusted odds revealed children presenting nasal obstruction had higher risk of ARI (AOR: 6.6, 95% CI: 1.65, 26.39), but chest pain was negatively associated with the detection of any of the pathogens of *FilmArray RP2plus* (AOR: 0.09, 95% CI: 0.01, 0.96).

**Table 3:** Relation between Symptoms and ARI.

		**ARI negative –/n (%)**	**ARI positive +/n (%)**	**COR (95% CI)**	***p*-value**	**AOR (95% CI)**
Time from first Symptoms	1–2 days	3/26 (11.5)	23/26 (88.5)			
	3–5 days	5/40 (12.5)	35/40 (87.5)	0.91 (0.20–4.20)	0.91	–
	6–7 days	19/63 (30.2)	44/63 (69.8)	0.30 (0.08–1.13)	0.08	–
Nasal obstruction	Absent	6/10 (60.0)	4/10 (40.0)			
	Present	21/119 (17.6)	98/119 (82.4)	7.00 (1.82–27.00)	<0.01	6.60 (1.65–26.39)^**^
Chest Pain	Absent	24/125 (19.2)	101/125 (80.8)			
	Present	3/4 (75.0)	1/4 (25.0)	0.08 (0.01–0.80)	0.03	0.09 (0.01–0.96)^*^
Conjunctivitis	Absent	23/123 (18.7)	100/123 (81.3)			
	Present	4/6 (66.7)	2/6 (33.3)	0.12 (0.02–0.67)	0.02	–
Cough	Absent	6/17 (35.3)	11/17 (64.7)			
	Present	21/112 (18.8)	91/112 (81.3)	2.36 (0.79–7.12)	0.13	–
Nu. of Typical Symptoms ARI	1	4/9 (44.4)	5/9 (55.6)	1.04 (0.60–1.79)	0.89	–
	2	7/25 (28.0)	18/25 (72.0)			
	3	12/87 (13.8)	75/87 (86.2)			
	4	2/6 (33.3)	4/6 (66.7)			
	5	1/1 (100.0)	0/1 (0.0)			
	7	1/1 (100.0)	0/1 (0.0)			

*COR, crude odds ratio; AOR, adjusted odds ratio; OR, odds ratio; CI, confidence interval*.

* *p < 0.05*;

** *p < 0.01*.

### Socio-Demographic Risk Factors for ARI

Information regarding several socio-demographic factors was also collected for all patients included in this study. To determine their association with ARI, the proportion of children with each potential risk factor were compared in ARI positive and negative groups (Table 4). The bivariable analysis revealed that age, household crowding, householder studies, householder income, residence regime, number of children <5 years old that sleeps with an adult and presence of domestic animals were variables with a *p* < 0.2, therefore were included in the multivariable logistic regression model.

**Table 4:** Risk factors for ARI.

**Risk factors**		**ARI negative –/n (%)**	**ARI positive+/n (%)**	**OR (95% CI)**	***p*-value**	**AOR (95% CI)**
Age	<1	9/60 (15.0)	51/60 (85.0)			
	1–4	18/69 (26.1)	51/69 (73.9)	0.50 (0.20–1.22)	0.13	n/a
Household crowding	<4	0/14 (0.0)	14/14 (100.0)	n/a	0.02	
	4–5	19/64 (29.7)	45/64 (70.3)			
	>5	8/51 (15.7)	43/51 (84.3)			
Householder studies	None	0/7 (0.0)	7/7 (100.0)	n/a	0.05	
	Basic	8/29 (27.6)	21/29 (72.4)			
	Secondary	10/68 (14.7)	58/68 (85.3)			
	Superior	9/25 (36.0)	16/25 (64.0)			
Householder income	<15,000$	5/24 (20.8)	19/24 (79.2)		0.16	
	15,000$-20,000$	8/45 (17.8)	37/45 (82.2)	1.21 (0.35–4.234)	0.76	-
	20,000$-40,000$	3/28 (10.7)	25/28 (89.3)	2.19 (0.47–10.34)	0.32	-
	>40,000$	11/32 (34.4)	21/32 (89.7)	0.50 (0.15–1.71)	0.27	-
Residence regime	Owner	14/55 (25.5)	41/55 (74.5)		0.17	
	Rent	9/35 (25.7)	26/35 (74.3)	0.99 (0.37–2.61)	0.98	-
	With other relatives	4/39 (10.3)	35/39 (89.7)	2.99 (0.90–9.91)	0.07	-
Nu. of children <5 years old that sleeps with an adult	0 children's	7/11 (63.6)	4/11 (36.4)		<0.01	
	1 child	9/85 (25.7)	26/85 (74.3)	8.17 (2.12–31.48)	<0.01	8.17 (2.12–31.48)^**^
	>1 child	4/33 (10.3)	35/33 (89.7)	9.80 (2.07–46.35)	<0.01	9.80 (2.07–46.35)^**^
Presence of domestic animals	No	20/77 (26.0)	57/77 (74.0)			
	Yes	7/52 (13.5)	45/52 (86.5)	2.26 (0.88–5.81)	0.09	-

*COR, crude odds ratio; AOR, adjusted odds ratio; OR, odds ratio; CI, confidence interval*.

** *p < 0.01, n/a: not applicable*.

Forward stepwise binary logistic regression models examined factors associated with ARI obtaining a model for the prevalence of ARI accounting for 12.4% (Nagelkerke *R*^2^ = 0.124) of the variance in prevalence. In the final model, solely the number of children <5 years old sleeping in the same room with an adult was significantly associated with ARI. Thus, children <5 years old sharing the bedroom with an adult increased significantly their risk of ARI (OR: 8.17, 95% CI: 2.12, 31.48) compared to those living in households where no children shared the bedroom with adults. This odds ratio was even higher (OR: 9.8, 95% CI: 2.07, 46.35) when more than one child shared the same bedroom with an adult (Table 4). Interestingly, other factors such as household crowding, family income or the level of studies of the head of the family did not increase the risk of ARI among children <5 years old in our study.

## Discussion

To the best of our knowledge, this study is the first to investigate the role of 21 respiratory pathogens in children <5 years attended at the pediatrics service at the Hospital Dr. Agostinho Neto (HAN) in Praia city, Santiago Island, Archipelago of Cabo Verde with suspected ARI. This study reveals the pathogen profile that causes ARI in children <5 years and describes some clinical and socio-demographic factors associated with these infections. Our results are relevant toward the design and implementation of control programs and the optimal utilization of scarce resources for the most effective preventive, early management strategies, aiming to reduce the rate of morbidity and mortality by ARI in Cabo Verde.

Our results show a high infection rate (%) in the population included in our study, in line with similar studies performed in different locations worldwide. For example, in Naples province (Southern Italy) the rate of ARI in children <5 years was 78.0% (25) and in Lusaka province in Zambia was 76.8% (28), however studies in Malaysia and Ghana revealed much lower infection rates (29, 30). The variations in the published results can be explained by the differences in socio-economic factors, geographic and climatic differences, enrollment criteria, as well as the efficiency of local health care systems in different countries and patients' age (7, 9, 25). Nevertheless, authors attribute testing platforms an important factor for the high rate and diversity of pathogens to be detected, due to the sensitivity and specificity of the testing platforms (9, 31).

In our data, viruses are the main causative agents of ARI in children <5 years with suspected ARI (76.7% of all reported cases), with HRV/EV, FluA H3 and RSV being the most prevalent. The overall prevalence is comparable to previous studies done in other areas, like Naples (25), China (8, 18), in three geographically distinct U.S. sites (26) and in Greece (32). All these highly prevalent viruses are usually associated with upper and lower respiratory tract infections to severe respiratory illness in infants, including bronchiolitis, pneumonia, wheezing, and are a leading cause of hospitalization in pediatrics emergency departments (18, 25, 32, 33). Cabo Verde does not have a surveillance system for agents that cause ARI, despite the high prevalence of reported cases of ARI in children <5 years. Therefore, our findings become important, because some of these viruses have also been described as responsible for infections in other organs such as heart and central nervous system (33), and these viruses can play a more significant role than originally thought. So additional studies describing our serotypes and/or variants will help to further understand how much these viruses contribute to severity of disease in this community and their role in infection dynamics at national and international level.

Co-infection with multiple respiratory pathogens was detected in 20.6% of the positive specimens tested by *FilmArray*® *Respiratory Panel v.2.0 Plus* in our study, in line with some reports in the world (28, 34). Interestingly, studies in patients with ARI suggest that pediatric patients are more likely to be infected by multiple pathogens than adults (8). We showed that HRV/EV were the most frequent pathogens detected in co-infection in children with suspected ARI, being HRV/EV+ADV the most frequent combination which is in line with reported data in asymptomatic and symptomatic children (8, 25, 32, 35). Although the reason behind co-infection between HRV/EV and other pathogens that cause ARI is unclear, the infection mechanisms used by this virus have been suggested as important factors that facilitate connection, translation and persistence of other viruses and bacteria (36).

Variations in the rates of influenza infections have been reported in several studies in different age groups, especially in children <5 years (37–40). In our study, FluA H3 was the only subtype of influenza virus showing a statistically significant difference by age, being more prevalent in children aged between 1 and 4 years than <1 year (*p* = 0.04). To explain this variation, the interaction between age and susceptibility to influenza infection have been suggested, as the immunity against influenza infection probably differs between age groups due to previous exposure to different influenza subtypes (41).

Interestingly, adenovirus was more common in female children than male children. In contrast, additional studies showed that male children are more susceptible to adenoviruses infections (42). To date, no study involving children <5 years with suspected ARI has reported a relationship between positive cases of ADV and sex (13, 42–45), and the risk factors for adenovirus respiratory infection in children are controversial (44). However, adenoviruses are considered important causative agents of ARI in children (13), accounting for 2–5% of the overall respiratory infections and 4–10% of the pneumonia (43, 45, 46).

Statistical differences in the positivity rates of ARI were noted across seasons in our study, with November and May being the months with highest positive rates of ARI. Among the pathogens tested, only hMPV, FluA H3, Flu B, and RSV showed a distinct seasonality, in line with other studies from Zambia,Kenya and Brazil (12, 13, 34, 47, 48). Seasonal fluctuations in the incidence of ARI caused by viruses/bacteria are associated with the climatic characteristics of the study areas (25, 49, 50). As an example, in tropical areas, more than one period of viral/bacterial activity can occur during the year, with peaks during the mild winter and rainy season; in contrast, in the temperate areas, the peaks occur mainly in winter (12, 13, 47, 51, 52). This phenomenon has been very well studied in southern Brazil, where researchers have found that adenoviruses circulate year-round, particularly in the summer (13), PIV 1 circulates in the fall, PIV 2 during fall and spring, and PIV 3 during spring (47). In contrast, influenza A and B circulate mainly in winter (12). Additional studies will be necessary to understand the seasonality of ARI caused by viruses/bacteria in Cabo Verde.

Atypical respiratory pathogens *Chlamydia pneumoniae, Mycoplasma pneumoniae* and *Legionella pneumophila* are recognized as a significant cause of ARI (53–55). Although *C. pneumonia* and *M. pneumoniae* were only found in low frequency in this study, it is important to monitor them in this community because previous studies demonstrated the implication of these bacteria in community-acquired pneumonia (CAP), acute exacerbations of chronic bronchitis, asthma, and less frequently, upper respiratory-tract infections (53, 56, 57).

Several Coronavirus (CoVs) subtypes have been described as causative agents of acute respiratory infections in humans since the mid-1960s (58). Four endemic CoVs subtypes were described in our study, CoV-229E, CoV-OC63, CoV-HL63, and CoV-HKU1, but all in low frequencies. It is well known that CoVs are implicated in cases of severe respiratory tract infections worldwide, and despite the growing interest, important gaps in the knowledge about CoVs remain (58–60).

Different clinical presentations have been associated with ARI worldwide (61–63). Despite the association found between several symptoms and ARI in our study, only the presence of nasal obstruction significantly increased the risk of ARI caused by any of the described pathogens. Evidence from other studies indicated an association between fever and an increased risk of severe respiratory infection (61, 64), but not for chest pain, cough, nasal obstruction and conjunctivitis (61, 65). Interestingly, children reporting chest pain had significantly decreased odds of ARI in or study. This finding can be explained by the fact that chest pain have been associated with several other etiologies, apart from ARI (66–68).

This study also described the social and demographic factors associated with ARI caused by any of the pathogens included in Film Array RP2 plus. In our data, only having one or more children sharing the bedroom with an adult increased significantly the risk among children <5 years attending the pediatrics service of Dr. Agostinho Neto Hospital in Praia city, Santiago Island, Cabo Verde with suspected ARI. Interestingly, other variables generally described as risks factors for ARI in children <5 years worldwide, such as age and household crowding, did not show that association in our model (69–71). In our study, the prevalence of ARI was equally distributed among the age groups, but additional studies show that children under 1 year of age are more susceptible to ARI compared to those aged >1 years (70, 71). Increased ARI among lower age children might be due to less developed immunity (72).

In conclusion, 17 different respiratory pathogens were detected as etiological agents for ARI in children <5 years attending at the pediatrics service at the Dr. Agostinho Neto Hospital (HAN) in Praia city, Santiago Island, Cabo Verde. Viruses were the main etiologic agents of ARI in this population, being HRV/EV, FluA H3, and RSV the most prevalent agents detected. Besides, some socio-demographic data and clinical symptoms are described as risk factors for ARI in children <5 years old. The data addresses the lack of studies on respiratory tract infections in Cabo Verde and highlights the need of more studies involving other health services in the country. However, these findings may be useful at national and international level. Moreover, future efforts to reduce the impact of ARIs will depend on the commitment of competent organs to mobilize resources to finance and implement programs to introduce vaccines, particularly in this age group where most of the morbidity and mortality is associated with ARI.

## Data Availability Statement

The original contributions presented in the study are included in the article/supplementary material, further inquiries can be directed to the corresponding author.

## Ethics Statement

The studies involving human participants were reviewed and approved by National Health Research Ethics Committee of Cabo Verde (CNEPS) (deliberation no. 72/2018). Written informed consent to participate in this study was provided by the participants' legal guardian/next of kin.

## Author Contributions

EC and IIMdP-A designed, supervised, oversaw the study implementation, and revised the manuscript. WC designed, implement the study, collected the data, interpreted the data, and wrote the manuscript. RD-G conducted the analysis of the data and revised the manuscript. MS and CA worked in sample and data collection at the pediatric service of Dr. Agostinho Neto Hospital. BV revised the manuscript. All authors have contributed substantially to the study, read, and approved the final manuscript.

## Funding

This study was funded by Fundación Canaria para el Control de Enfermedades Tropicales (FUNCCET), Cabildo de Tenerife Proyectos de Cooperación e Investigación and Instituto Universitario de Enfermedades Tropicales y Salud Publica de Canarias en Universidad de La Laguna (IUETSPC/ULL) Tenerife, Islas Canarias, Spain.

## Conflict of Interest

The authors declare that the research was conducted in the absence of any commercial or financial relationships that could be construed as a potential conflict of interest.

## Publisher's Note

All claims expressed in this article are solely those of the authors and do not necessarily represent those of their affiliated organizations, or those of the publisher, the editors and the reviewers. Any product that may be evaluated in this article, or claim that may be made by its manufacturer, is not guaranteed or endorsed by the publisher.

## Abbreviations

RICET: Red de Investigación Colaborativa en Enfermidades Tropicales
ARI: Acute Respiratory infection
ADV: Adenovirus
CoV 229E: Coronavirus 229E
CoV HKU1: Coronavirus HKU1
CoV OC43: Coronavirus OC43
CoV NL63: Coronavirus NL63
HRV/EV: Human Rhinovirus/Enterovirus
hMPV: Human Metapneumovirus
FluA: Influenza A
Flu A H1 2009: Influenza A H1 2009
Flu A H3: Influenza A H3
Flu A H1, Influenza A H1, Flu B: Influenza B
PIV 1: Parainfluenza Virus 1
PIV 2: Parainfluenza Virus 2
PIV 3: Parainfluenza Virus 3
PIV 4: Parainfluenza Virus 4
RSV: Syncytial Respiratory Virus
MERS-CoV: Middle East Respiratory Syndrome
SARS-CoV: Severe Acute Respiratory Syndrome Coronavirus
SARS-CoV-2: Severe Acute Respiratory Syndrome Coronavirus 2
hBoVs: Human bocavirus
*C. pneumoniae*: *Chlamydia pneumoniae*
*M. pneumoniae*: *Mycoplasma pneumoniae*
OSMF: OpenStreetMap Foundation
NPS: Naso-pharyngeal
VTM: Viral Transport Medium
IUETSPC/ULL, Instituto Universitario de Enfermedades Tropicales y Salud Pública de Canarias: Universidad de La Laguna
PCR: Polymerase Chain Reaction
FARP: FilmArray® Respiratory Panel
COR: Crude odds ratios
AOR: Adjusted odds ratios
CAP: Community-acquired pneumonia
CoVs: Acute Several Coronavirus
CNEPS: National Health Research Ethics Committee of Cabo Verde
FUNCCET: Fundación Canarias para el Control de Enfermidades Tropicales
INSP: National Institute of Public Health of Cabo Verde.
